# Effect of high and low-supportive footwear on female tri-planar knee moments during single limb landing

**DOI:** 10.1186/s13047-018-0294-x

**Published:** 2018-09-10

**Authors:** Timothy A. Sayer, Rana S. Hinman, Kade L. Paterson, Kim L. Bennell, Karine Fortin, Adam L. Bryant

**Affiliations:** 1 Centre for Health Exercise & Sports Medicine, Department of Physiotherapy, The University of Melbourne, 161 Barry St, Melbourne, 3052 Australia; 2 Exercise and Sports (LUNEX), Grand-Duchy of Luxembourg, International University of Health, Differdange, Luxembourg

**Keywords:** Footwear, Kinetics, Adolescent, Landing

## Abstract

**Background:**

Higher landing-related external knee joint moments at later stages of female pubertal development likely contribute to a higher incidence of non-contact anterior cruciate ligament (ACL) injury. Athletic footwear may provide a potential strategy to alter higher knee moments.

**Methods:**

Thirty-one late/post-pubertal girls (Tanner stage IV-V, menarche and growth spurt attained) performed a single limb drop lateral jump in three footwear conditions (barefoot, low support shoes and high support shoes), in which peak knee abduction moment (KAbM), flexion moment (KFM) and internal rotation moments (KIRM) were measured. Repeated measures ANOVA and ANCOVA were used to test for a main effect of footwear with and without foot posture index (FPI) as a covariate (*p* < 0.05) with *post-hoc* test carried out via Fisher’s Least Significant Difference (LSD).

**Results:**

A main effect of footwear condition was observed for peak KFM (*p* < 0.05), but not KAbM or KIRM, in both unadjusted and adjusted models. *Post-hoc* analysis demonstrated that both high- and low-support shoes increased peak KFM compared with barefoot (*p* < 0.001*).*

**Conclusion:**

Our findings indicate commercially available high- and low-supportive footwear increase peak KFM, but do not effect KAbM or KIRM while landing among late/post-pubertal girls. This suggests that these styles of footwear are inadequate at reducing higher knee moments in an at-risk cohort.

## Background

A high prevalence of anterior cruciate ligament (ACL) injury amongst adolescent girls aged 14–18 years old is multifactorial [1]; however, hormonal and biomechanical factors are emerging as primary contributors [2–4]. Adolescence is an important stage in the context of female musculoskeletal development, as it typically involves a rapid influx of estrogen and growth factors, commonly referred to as puberty. Considering puberty involves an increase in height, muscle cross sectional area and overall body mass [5], it’s suspected that these physical characteristics may indeed lead to aberrant lower limb biomechanics related to adolescent female non-contact ACL injury. Specifically, higher external tri-planar knee joint moments, such as the knee abduction moment (KAbM), flexion moment (KFM) and internal rotation moment (KIRM) contribute to a tri-planar mechanism of ACL rupture [6]. More importantly, it appears that girls at later stages of pubertal development (i.e., late/post-pubertal development) exhibit higher barefoot landing-related knee moments [7–9], which may predispose them to a higher risk of injury [10], highlighting a need for strategies that ameliorate these peak knee moments.

In reality, barefoot participation is impractical for most sports relevant to non-contact ACL injury and, as such, girls wear various types of athletic footwear during sports participation. Surprisingly, there are no published cohort-specific (i.e. puberty) studies investigating the effects of footwear on knee biomechanics. However, previous research involving both healthy [11–15] and populations with knee pathology (e.g. older adults with knee osteoarthritis) [16] have demonstrated a knee load-modifying role of footwear. Specifically, modifying medial and lateral support features of a shoe can influence the resultant frontal (i.e., KAbM) and transverse plane (i.e., KIRM) moments [11–16] which may be important in the context of ACL injury, given that combined valgus and rotation increases ACL strain [17]. The athletic footwear market contains a wide variety of both “high-supportive” and “low-supportive” footwear options. High-supportive shoes commonly feature a medial post, increased longitudinal shoe stiffness and midfoot rotational stability to minimize excessive foot pronation during activity, whilst low-supportive shoes do not contain such features and allow natural pronation to occur [18].

It is thought that the biomechanical effects of excessive foot pronation during single-limb landing are transferred up the kinetic chain in female athletes, contributing to elevated KAbM and KIRM [17, 19, 20]. To counteract these loads, high-supportive shoes with appropriate anti-pronation features [18], might potentially reduce KAbM and/or KIRM. In support, footwear studies investigating the effects of wedges/insoles and orthotics on knee biomechanics [13–15, 20], have demonstrated their influence on knee loads by altering the frontal plane position of the knee relative to the resultant ground reaction force vector [21, 22]. Specifically, laterally arched/wedged insoles provide a laterally-directed (eversion) bias to the foot shifting the centre of pressure laterally and increasing the KAbM [22]. By contrast, medially arched/wedged insoles create a foot inversion bias, shifting the centre of pressure medially and lowering the KAbM [21]. Furthermore, a medial arch support also has the capacity to externally rotate the tibia relative to the femur, thereby limiting internal tibial rotation and the potential magnitude of KIRM during foot-ground contact [23]. Hence, it’s plausible that high-supportive athletic footwear could attenuate peak KAbM and KIRM during single-limb landing relative to low-supportive footwear and barefoot conditions.

Arguably, athletic footwear also has the potential to increase the risk of non-contact ACL injury by increasing peak KFM during sporting activities. Many athletic shoes possess a raised heel with respect to the forefoot (i.e. pitch) – a feature that has been previously shown to increase peak KFM compared to barefoot in adult runners [11, 12, 24]. Given that high-supportive shoes often have an increased pitch compared to their low-supportive counterparts, it’s imperative that an examination of high-support footwear includes an analysis of frontal, transverse and sagittal plane knee moments (i.e., KAbM, KIRM and KFM) as many late/post-pubertal girls are likely wearing these types of shoes while playing sport.

Therefore, the aim of this study was to investigate whether a difference in tri-planar knee moments exist between high-support shoes, low-support shoes and barefoot in late/post-pubertal girls. Our primary hypothesis was that the high-supportive shoe would exhibit lower peak KAbM and KIRM compared to both the low support shoes and barefoot. In contrast, our secondary hypothesis was that both high- and low- support shoes would increase peak KFM compared to barefoot.

## Methods

### Participants

Participants in this study were a sub-group of a wider study investigating biomechanics across stages of female pubertal development [25]. A total of 31 girls were recruited from the University of Melbourne campus, local schools, community centres and sporting facilities with all girls required to pass inclusion criteria: (i) female; (ii) aged 14–25 years old; (iii) participating in regular physical activity; and, (iv) healthy weight (BMI < 30 kg/m^2^). Exclusion criteria were: (i) history of lower limb injury, knee pain or medical condition affecting walking, running and jumping (ii) previous ACL or meniscal injury or (iii) bi-or tri-phasic oral contraceptive pill (OCP) use. The decision to exclude girls using a bi- or tri-phasic OCP was to limit the influence estrogen could have on lower limb biomechanics, as these types of pills produce fluctuations in estrogen across the menstrual cycle [3]. All participants, together with parents/guardians of those < 18 years of age, signed an informed consent form. This study was supported by an Australian Research Council linkage grant (LP150101041) with ethics approved by the University of Melbourne Human Research Ethics Committee (#1442604).

Following eligibility, all 31 girls were classified as late/post-pubertal via a modified pubertal assessment using the Tanner stages of breast development, questions regarding their menstrual cycle and adolescent growth. Specifically, Tanner staging was based upon self-rated breast development via an online, de-identified questionnaire containing pictures and modified diagrams [26, 27]. The Tanner stages have demonstrated appropriate levels of validity and reliability in a cohort of adolescent females > 12 years old [28, 29]. The questionnaire also ascertained if the adolescent growth spurt had occurred (yes or no response) using the question “The adolescent has grown 3-3.5 inches (7.5-9cm) in the past 6 months or is past this growth spurt” and whether menarche had commenced via the statement “The adolescent has begun menarche (period)” taken from the modified pubertal maturation observational scale [8, 30]. To be classified as late/post-pubertal, girls indicated they were between Tanner stage IV-V and answered “yes” to both menarche and adolescent growth questions. Girls who did not meet the above criteria were excluded from the study.

### Estradiol and menstrual cycle control

Considering puberty involves a rapid rise in estrogen [3], each participant was tested while estrogen levels were low, as higher levels could influence our external knee joint moment measures [3]. Given that all girls had regular menstrual cycles but may have been using a monophasic OCP, different strategies were employed to ensure low levels of estrogen at time of testing. Eumenorrheic girls were required to identify their early follicular phase (days 1–7 following menses) at which time biomechanical testing was performed. In contrast, monophasic OCP users were tested at anytime, given consistency of estradiol levels. To ensure that all girls were indeed tested with low levels of estrogen, a 5 mL saliva sample was collected immediately before testing. Samples were then analyzed via enzyme immunoassay according to the manufacturer’s instructions (Nutripath Integrative Pathology, Melbourne, Australia). All girls were required to have estradiol concentration levels < 18 pmol/L according to the reference ranges for the follicular phase provided by the manufacturer.

### Participant characteristics

Following saliva collection, descriptive measures of height and weight were recorded while barefoot. Static foot posture was recorded by the primary investigator using the foot posture index (FPI; [31]) on the non-dominant limb determined via the Lateral Preference Inventory (LPI; [32]). We used the non-dominant limb given that previous literature indicates higher female ACL injury rates on this limb [33] and recorded foot posture given that foot pronation has been suggested to affect knee biomechanics associated with ACL injury [19]. The primary investigator was trained by an experienced podiatrist with extensive use of the FPI [31]. All participant descriptive measures are summarized in Table 1.

**Table 1:** Descriptive characteristics

Variable	Late/post-puberty
*n*	31
Age (years)	19.8 ± 4.0
Weight (kg)	60.5 ± 8.5
BMI	21.9 ± 3.1
Height (m)	1.7 ± 0.1
Estradiol (pmol/L)	9.5 ± 5.1
Thigh Segment Length (cm)	43.7 ± 2.1
Shank Segment Length (cm)	38.8 ± 2.1
Foot Posture Index (value)	2.5 ± 3.6
Normal (n, %)	55
Supinated (n, %)	19
Pronated (n, %)	26

All variables are reported as mean (SD). Foot Posture Index categories are also provided for the cohort

### Footwear classification

**Fig. 1:**
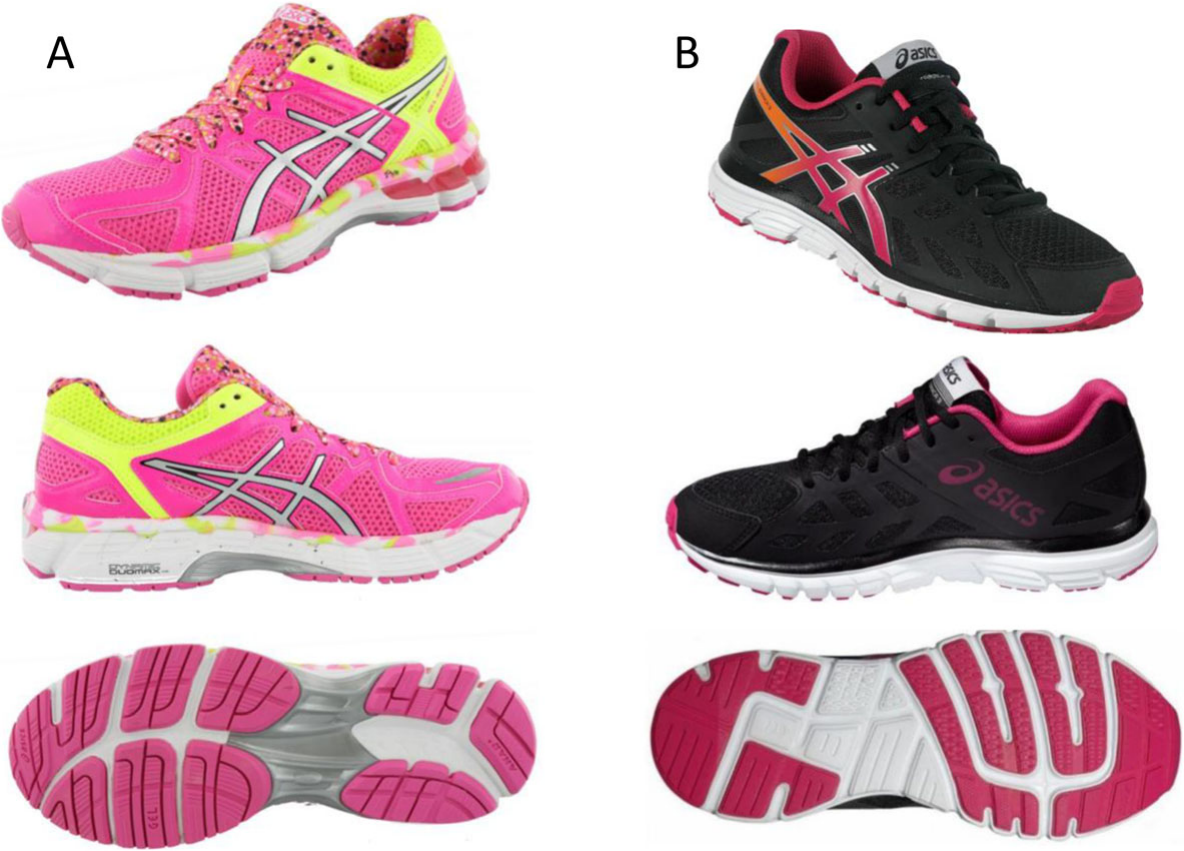
The high-support shoes (ASICS Kayano-GS, **a**) featured a medial post, < 10° torsional stiffness, < 10° heel counter stiffness and < 45° midfoot longitudinal stability. In contrast, the low-supportive shoes (ASICS Zaraca 3, **b**) featured a uniform midsole density, 10–45 ° torsional stiffness, 10–45° heel counter stiffness and > 45° midfoot longitudinal stability

Currently no definition exists to designate footwear as high- or low-support. To categorize our shoes, we consulted recommendations from the Footwear Assessment Tool (FAT; [18, 34]) for key footwear features. Subsequently, we developed a set of criteria a priori to ensure that our high-support footwear featured elements we thought would reduce KAbM and KIRM. These features included (i) a midsole with a higher density/stiffness medially compared to laterally (e.g. medial post), (ii) < 10° torsional stiffness, (iii) < 10 degrees heel counter stiffness and (iv) < 45° midfoot longitudinal stability. By contrast, the low-support footwear characteristics included: (i) uniform midsole density (e.g. no medial post), (ii) 10–45° heel counter stiffness (iii) 10–45° torsional stiffness, (iv) > 45° midfoot longitudinal stiffness. As a result, we selected the Asics Kayano-GS as our high-support shoe and the Asics Zaraca as our low-support shoe (Fig. 1). Both shoes were the current model at time of testing.

The presence of a medial post and midsole density was determined by the manufacturer. Longitudinal shoe stiffness was assessed by bending the midfoot of the shoe in the sagittal plane and rating as moderate rigidity (shoe bends < 45°) or mild (shoe bends > 45°). Torsional stiffness was subjectively assessed by rotating the midfoot of the footwear to broadly classify the stiffness as rigid (< 10° rotation) or moderate (10–45°). Likewise, heel counter stiffness was determined as per the FAT [18], where the heel counter was clasped with the index finger and thumb approximately 20 mm from its base and squeezed in the frontal plane to estimate the angular displacement.

Further technical information related to the high support shoes (Asics Kayano –GS) included: (i) heel stack height = 25 mm, (ii) forefoot stack height = 12 mm, (iii) footwear pitch = 13 mm and (iv) shoe mass = 260 g. For the low support shoes (Asics Zaraca 3) these features included: (i) heel stack height = 28 mm, (ii) forefoot stack height = 18 mm, (iii) footwear pitch = 10 mm and (iv) shoe mass = 240 g.

### Landing task

**Fig. 2:**
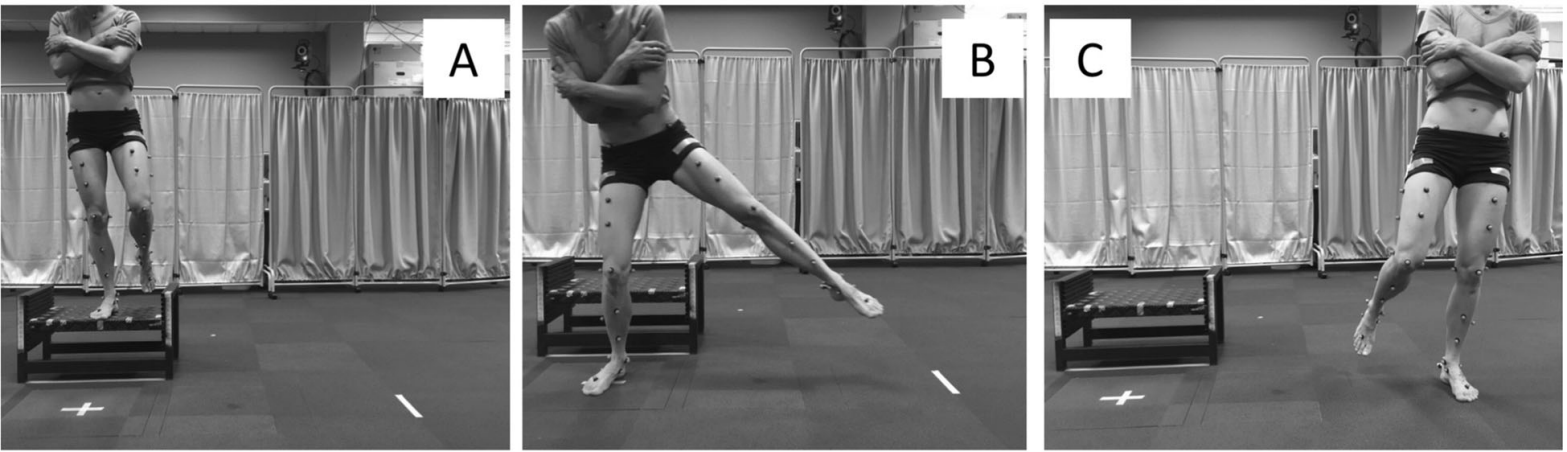
The single-limb drop lateral jump task involved the participant balancing on their non-dominant test limb with hands folded across chest (**a**), hop down towards the “X” marked on the ground (**b**) and then laterally hop/cut 90° as quickly as possible towards their dominant limb, landing and balancing for 5 s (**c**)

Before each participant was familiarized with the landing task, the primary investigator affixed reflective markers to each participant’s trunk, thigh, shank and foot according to a model previously described by Schache and Baker [35]. All participants were then familiarised with a single limb drop lateral jump (DLJ, Fig. 2). For each participant, jump height was scaled to a relative box height of 30% of their lower limb length, measured from the outermost lateral aspect of the greater trochanter to the floor. We normalized box height to individual limb anthropometry, to create similar neuromuscular demand between participants, as an individual’s jump/landing height increases during adolescent years [36].

For each footwear condition (barefoot, low-support and high-support shoes), participants aligned their foot to the centre of the box with hands folded across their chest and then hopped forwards from their test limb (non-dominant leg) and upon landing, immediately hopped/cut 90° towards the opposite limb, balancing for 5 s on a marker placed on the ground at a distance of 150% lower limb length from the center of the force plate (Fig. 2). Three successful trials were performed in each of the three different footwear conditions: i) barefoot, ii) low-support and, iii) high-support shoes. Testing order of footwear was pre-determined via block randomization for each participant (i.e. 1 block: 1 pubertal groups × 3 footwear conditions).

### Motion and data analysis

Kinematic (120 Hz) and GRF data (2400 Hz) were collected using a 12-camera Vicon motion analysis system (Oxford, UK) synchronized with a concealed force plate (AMTI, Inc., Watertown, MA, USA). Data were filtered using a fourth order zero-lag Butterworth filter at a frequency of 20 Hz. Joint moments were calculated using inverse dynamics and were expressed in the distal anatomical reference frame and normalised to body mass (N·m/kg; [35]). Negative values indicate higher KAbM and KIRM and positive values indicate higher KFM. Peaks for all moments were derived and exported for analysis. Joint moments were evaluated during the first 25% of stance as ACL injury typically occurs shortly after initial contact [4]. Anthropometric segment lengths for the thigh and shank were extracted from the kinematic model.

All data are reported as the mean and standard deviation (SD) with the mean difference (MD) and 95% confidence intervals reported for significant variables. Repeated measures ANOVA and ANCOVA were run for peak KAbM, KFM and KIRM. We performed statistical comparisons for peak knee moments across footwear conditions with and without FPI as a covariate to determine whether it had any effect. Where significant, *post-hoc* analysis was performed using Fisher’s Least Squares Difference (LSD) tests. All data were analysed using the Statistical Packages for Social Science (SPSS, Version 23, IBM) and significance was set at 0.05.

## Results

For the frontal plane KAbM and transverse plane KIRM, no differences were found for all comparisons in either unadjusted (*p* > 0.05) or adjusted analyses (*p* > 0.05, Table 2). However, for the peak KFM, there was a main effect for both adjusted and unadjusted analyses (*p* < 0.001). *Post-hoc* results revealed that both adjusted and unadjusted results were identical, with the high-support shoe exhibiting higher peak KFM (MD = 0.44, 95% CI 0.36 to 0.53 N·m/kg, *p* < 0.001, Table 2) than the barefoot condition. Likewise, results for the low-support shoe were identical for both adjusted and unadjusted FPI whereby higher peak KFM (MD = 0.36, 95% CI 0.25 to 0.48 N·m/kg, *p* < 0.001) was observed compared to barefoot. For the between shoe comparison, there was a trend indicating higher KFM in the high-support compared to low-support shoe for both adjusted (*p =* 0.053) and unadjusted (*p* = 0.050) analyses.

**Table 2:** Peak tri-planar knee moments for each footwear condition

	KAbM (N·m/kg)		KFM (N·m/kg)		KIRM (N·m/kg)	
	Unadjusted	Adjusted^1^	Unadjusted	Adjusted^1^	Unadjusted	Adjusted^1^
Barefoot	−0.43 ± 0.18	−0.43 ± 0.18	2.90 ± 0.42	2.85 ± 0.43	−0.23 ± 0.11	−0.23 ± 0.11
High-support	− 0.41 ± 0.20	−0.41 ± 0.21	3.3 ± 0.39 ^*a*^	3.3 ± 0.39 ^*a*^	−0.25 ± 0.11	−0.25 ± 0.11
Low-support	− 0.44 ± 0.16	−0.47 ± 0.16	3.22 ± 0.41^*a*^	3.22 ± 0.41^*a*^	−0.23 ± 0.09	−0.23 ± 0.09

Results are presented as mean (SD). Negative values represent a larger peak KAbM and KIRM, whereas for the KFM, positive values indicate a larger peak moment. Both unadjusted and adjusted values are reported for FPI as a covariate in the statistical model

*a* denotes significantly different to barefoot condition (*p* < 0.001)

1 denotes statistical comparison via ANCOVA, correcting for the influence of FPI

## Discussion

This is the first study to compare the effects of different footwear conditions on tri-planar knee moments in a cohort of girls classified as late/post-pubertal development during a single-limb landing task. Our findings reject our primary hypothesis, as the high-support shoe did not ameliorate peak KAbM and KIRM compared to low-support and barefoot conditions. However, as expected, we confirmed our secondary hypothesis that both shoe types would increase peak KFM compared to barefoot. Given these findings, it appears that both high- and low-supportive styles of footwear are inadequate at reducing peak KAbM and KIRM and in fact increase peak KFM, which may be detrimental for reducing the risk of non-contact ACL injury in this cohort [6].

Regarding the primary hypothesis related to both KAbM and KIRM, results from this study extend those reported by Bisesti and colleagues [37], who reported no difference in KAbM between barefoot and low support shoes in a mixed cohort of adult participants who performed a single limb 45° cutting task. However, findings from the present study are in contrast to previous studies investigating the effect of orthotics during single limb landing [38] and medial post/wedges during double limb drop vertical jumps [14, 39]. Specifically, two studies by Joseph and colleagues [14, 39], highlight that knee valgus, foot pronation and hip adduction are all reduced when female college athletes wore a 5° medial post in their shoes compared to no post. In light of these findings, the supportive features incorporated within the high-support shoes tested in this study may be inadequate to substantially influence frontal and transverse plane knee moments, and increasing the medial post of the shoes (e.g. addition of a medial wedge/orthotic) could potentially ameliorate knee moments.

Furthermore, adjusting for FPI did not affect either frontal or transverse plane loading results. As the average FPI in this cohort was 2.5 ± 3.6 which is categorised as a normal foot posture, it may be that the high- and low-support shoe effects on moments were diminished and may be more pronounced in pronated foot types. Indeed, support for this theory comes from a recent randomized controlled trial investigating the effect of standard and motion control footwear in runners, that demonstrated lower injury risk in runners who wore the motion control shoe and had a higher FPI score (i.e. pronated foot; [40]). Therefore, we recommend further FPI sub-group analysis be conducted in the future.

We confirmed the secondary hypothesis of higher peak KFM (≈10–12%) when landing in shoes compared to barefoot. Although we did not explore the mechanism by which shoes elevate peak KFM, elevated pitch wearing shoes compared to barefoot is a likely contributor [15]. For instance, we speculate that higher pitch (i.e., shoes) simultaneously reduces the sagittal plane ankle excursion while increasing the knee flexion angle during the stance phase of landing. Consequently, this may lead to a larger sagittal plane knee joint moment arm and higher peak KFM. In support, previous drop-landing studies have demonstrated a 22% increase in knee flexion angle at initial contact and a gradual increase in peak knee flexion angle with increasing heel heights (i.e. pitch; [15]). Moreover, despite the biomechanics of running and jumping being quite different, previous running-related research demonstrates approximately 18% lower peak KFM when running barefoot (i.e., lower pitch) compared to supportive athletic footwear (i.e., higher pitch; [12]). Hence, the present study in combination with the aforementioned previous running and landing studies provide evidence for future studies to examine if modifying footwear features can ameliorate peak tri-planar knee moments.

While this study provides new insights into the effect of athletic footwear of late/post-pubertal girls, several limitations should be acknowledged. Firstly, a cross-sectional study design does not answer whether different types of footwear influence knee moments over time. We speculate that an adaptation period may be required for participants to become accustomed to different types of footwear, which may ultimately lead to neuromuscular changes that result in lower peak knee moments, rather than instantaneous change measured in our study. Secondly, footwear was selected based on criteria outlined in previous literature [18], yet there is the potential limitation of extrapolating results to other footwear styles, or similar styles from other footwear manufacturers. Finally, both adjusted and unadjusted FPI values were reported to determine if FPI influenced results, yet we do not know whether FPI sub-types, particularly those with a pronated foot, are influenced to a further extent than supinated counterparts while wearing different shoes.

## Conclusion

High-support and low-supportive shoes do not ameliorate peak KAbM or KIRM compared to barefoot during single limb landing. In fact, both shoes exhibit higher peak KFM compared to barefoot which may be detrimental in the context of non-contact ACL injury prevention. Specifically, we recommend further research determines which footwear features are more likely to reduce knee moments during landing of pubertal girls.

## Abbreviations

ACL: Anterior cruciate ligament
ANCOVA: Analysis of covariance
ANOVA: Analysis of variance
BMI: Body mass index
CI: Confidence interval
FAT: Footwear assessment tool
FPI: Foot posture index
KAbM: Knee abduction moment
KFM: Knee flexion moment
KIRM: Knee internal rotation moment
LPI: Lateral preference
LSD: Least square’s difference, inventory
MD: Mean difference
OCP: Oral contraceptive pill
